# Effect of n-3 PUFA on extracellular matrix protein turnover in patients with psoriatic arthritis: a randomized, double-blind, placebo-controlled trial

**DOI:** 10.1007/s00296-021-04861-z

**Published:** 2021-04-22

**Authors:** Signe Holm Nielsen, Samra Sardar, Anne Sofie Siebuhr, Annette Schlemmer, Erik Berg Schmidt, Anne-Christine Bay-Jensen, Morten A. Karsdal, Jeppe Hagstrup Christensen, Salome Kristensen

**Affiliations:** 1 Immunoscience, Nordic Bioscience, Herlev Hovedgade 207, 2730 Herlev, Denmark; 2 Department of Biotechnology and Biomedicine, Technical University of Denmark, Lyngby, Denmark; 3 Department of Rheumatology, Aalborg University Hospital, Aalborg, Denmark; 4 Department of Cardiology, Aalborg University Hospital, Aalborg, Denmark; 5 Department of Clinical Medicine, Aalborg University, Aalborg, Denmark; 6 Department of Nephrology, Aalborg University Hospital, Aalborg, Denmark; 7 Department of Clinical Medicine, Aalborg University, Aalborg, Denmark

**Keywords:** Psoriatic arthritis, Collagens, Biomarkers, Fish oil

## Abstract

Psoriatic arthritis (PsA) is a chronic inflammatory disease characterized by involvement of skin, axial and peripheral skeleton. An altered balance between extracellular matrix (ECM) formation and breakdown is a key event in PsA, and changes in ECM protein metabolites may provide insight to tissue changes. Dietary fish oils (n-3 PUFA) might affect the inflammation driven tissue turnover. The aim was to evaluate ECM metabolites in patients with PsA compared to healthy individuals and investigate the effects of n-3 PUFA. The 24-week randomized, double-blind, placebo-controlled trial of PUFA included 142 patients with PsA. Fifty-seven healthy individuals were included for comparison. This study is a sub-study investigating biomarkers of tissue remodelling as secondary outcomes. Serum samples at baseline and 24 weeks and healthy individuals were obtained, while a panel of ECM metabolites reflecting bone and soft tissue turnover were measured by ELISAs: PRO-C1, PRO-C3, PRO-C4, C1M, C3M, C4M, CTX-I and Osteocalcin (OC). C1M, PRO-C3, PRO-C4 and C4M was found to be elevated in PsA patients compared to the healthy individuals (from 56 to 792%, all *p* < 0.0001), where no differences were found for OC, CTX-I, PRO-C1 and C3M. PRO-C3 was increased by 7% in patients receiving n-3 PUFA after 24 weeks compared to baseline levels (*p* = 0.002). None of the other biomarkers was changed with n-3 PUFA treatment. This indicates that tissue turnover is increased in PsA patients compared to healthy individuals, while n-3 PUFA treatment for 24 weeks did not have an effect on tissue turnover. Trial registration NCT01818804. Registered 27 March 2013–Completed 18 February 2016. https://clinicaltrials.gov/ct2/show/NCT01818804?term=NCT01818804&rank=1

## Background

Psoriatic Arthritis (PsA) is a chronic, inflammatory arthropathy of the peripheral and axial skeleton. PsA, in general, remains poorly defined because of its heterogeneous clinical presentation [1]. Pathologically, it is an autoimmune disease characterized by inflammation of the joints, entheseal sites and skin [2]. A major cause of disability in PsA is the destruction of the extracellular matrix (ECM) of cartilage, bone, and soft tissues of the joint [3, 4]. This is attributed to effects of pro-inflammatory cellular infiltrate and elaboration of inflammatory cytokines underlying the pathology of PsA. The inflammatory joint milieu induces increased expression of proteolytic enzymes, such as the matrix metalloproteinases (MMPs), That mediate enhanced turnover of the ECM [5, 6]. These ECM fragments may enter circulation and may be utilised as serological biomarkers of ECM turnover.

Degradation and formation of ECM proteins are in a tight equilibrium ensuring tissue health and homeostasis. This equilibrium is perturbed by the inflammatory load in PsA patients leading to altered balance of the ECM [7]. During this process, excessive levels of collagen fragments (neo-epitopes) are released into the circulation and may act as important biomarkers in the study of tissue-related remodelling. These biomarkers can also provide insight into identification of key molecular pathways leading to enhanced connective tissue remodelling.

The ECM can be divided into the basement membrane and interstitial matrix. Collagen types I and III are the most abundant collagens in soft tissue and are located in the interstitial matrix. Collagen type I is therefore predominant in both bone matrix and tissue. Collagen type III is found extensively in connective tissues, such as skin, and vascular system, and is found to be a key player in wound healing together with collagen type I [8, 9]. Collagen type IV is the main collagen component of the basement membrane, underlying epithelial or endothelial cells functioning as barrier between tissue compartments [10]. Besides soft tissues, the bone remodelling processes underlying PsA pathology can also be assessed objectively by the use of serological biomarkers. Here, the C-terminal telopeptide of collagen type I (CTX-I) measures the degradation of collagen type I by cathepsin K, an enzyme secreted by osteoclasts during bone resorption [11]. Several studies have established CTX-I as a specific and sensitive biomarker of bone resorption [12]. A second marker of bone turnover is osteocalcin (OC), which is one of the major non-collagenous proteins of bone matrix synthesized by active osteoblasts [13]. OC is a well-established markers of bone formation, and are found to correlate with histomorphometric bone formation indices [13, 14]. Increased bone remodelling and elevated levels of both degradation and formation markers have been documented in PsA patients previously [15]. An example of this was presented in 2015, where biomarkers of collagen type I degradation (C1M) and collagen type III degradation (C3M) were increased in PsA patients compared to healthy individuals.

The outlook of PsA has significantly changed in the past decades due to availability of various treatment options including conventional and biological disease-modifying antirheumatic drugs (DMARDs) and the recently released targeted synthetic disease-modifying antirheumatic drugs [16]. However, while other types of arthritis have seen significant improvements in outcomes with newer treatments, approximately half of the PsA patients still experience an insufficient response [17, 18].

Marine n-3 polyunsaturated fatty acids (PUFAs), such as eicosapentaenoic acid (EPA; 20:5n-3) and docosahexaenoic acid (DHA; 22:6n-3), found in fish and fish oils, have been proposed as potential therapeutic or supplementary agents in a variety of acute and chronic inflammatory settings. At sufficiently high intakes, n-3 PUFAs can act as anti-inflammatory agents by decreasing the production of arachidonic acid–derived inflammatory eicosanoids like prostaglandins and leukotrienes, pro-inflammatory cytokines, reactive oxygen species and the expression of adhesion molecules [19, 20]. Evidence of their clinical benefit is reasonably strong in some settings (eg, in RA [21, 22], cardiovascular disease [23], neurological diseases [24–26] and osteoarthritis [27, 28]) and weak in others (e.g., inflammatory bowel diseases and asthma). Recent randomized and controlled trial using n-3 PUFA has demonstrated a reduction in joint pain and non-steroidal anti-inflammatory drugs NSAIDs use in patients with PsA and RA [20, 29]. The clinical efficacy of n-3 PUFAs in arthritis can be attributed to their effect on inflammation and/or bone remodelling. Both mechanisms have been addressed individually in in vivo and in vitro setups [30–34]. However, the relative contribution of these mechanisms in various types of arthritis remains unexplored.

Biomarkers of ECM turnover can provide this valuable information regarding mechanism of action of n-3 PUFAs in arthritis. In this study, we aimed to explore the hypothesis that ECM turnover described by neo-epitope biomarkers can separate PsA patients from healthy individuals and patients treated with n-3 PUFA from the placebo group.

## Methods

### Study design and population

A randomized, double-blind, placebo-controlled trial was conducted and the subjects, study design, inclusion and exclusion criteria, randomization and sample size calculation has been described in details previously [29]. For 24 weeks, patients were assigned to a daily intake of either 3 g of marine n-3 PUFA (50% EPA and 50% DHA) or 3 g of olive oil (approximately 80% of oleic acid and 20% linoleic acid). Treatment with biological DMARDs or oral corticosteroids was exclusion criterion. Conventional DMARDs, NSAID and paracetamol intake were allowed.

All participants gave their written informed consent and the regional ethics committee of Northern Denmark approved the study (reference number N20120076). The study was conducted in accordance with the Declaration of Helsinki and registered at ClinicalTrials.gov (NCT01818804). Good Clinical Practice (GCP) inspectors monitored the study, and the GCP ethical and scientific quality requirements were fulfilled. The primary outcome of the study was disease activity measured by tender and swollen joint count [29]. This study is a sub-study investigating biomarkers of tissue remodelling as secondary outcomes. Blood samples were obtained from the study participants, at baseline and at the study end, in non-fasting state. Serum samples were collected and processed immediately after collecting according to standard operating procedures and stored at − 80 °C until analysis. For this sub-study, fifty-seven healthy individuals were included as controls, randomly selected and purchased from Lee Biosolutions (CA, US). Serum samples from the healthy individuals were collected in 2018 and processed immediately after collecting according to standard operating procedures and stored at − 80 °C until analysis.

### Biomarker analysis

ECM remodelling was assessed by a panel of biomarkers including validated ELISAs of Matrix-metalloproteinase-generated neo-epitope fragments of collagen type I, III and IV was chosen to assess collagen degradation (C1M, C3M, and C4M, respectively) and formation (PRO-C1, PRO-C3 and PRO-4, respectively) were run by manufactures instruction. Bone remodelling was assessed by osteocalcin (OC) and CTX-I. OC was measured using the Elecsys^®^ osteocalcin kit (Roche Diagnostic Ltd., Switzerland), while CTX-I was measured by the Elecsys^®^ β-CrossLaps assay (Roche diagnostics Ltd., Switzerland). Reference levels for OC and CTX-I were obtained from the package insert of the individual assays (*n* = 6). The biomarkers are described in Table 1.

**Table 1:** Overview of measured biomarkers representing ECM turnover

Biomarker	Measures	Reference
Collagen 2 turnover		
PRO-C1	Formation of collagen type I	[43]
PRO-C3	Formation of collagen type III	[44]
PRO-C4	Formation of collagen type IV	[45]
C1M	MMP-2, -9, -13 mediated degradation of collagen type I	[46]
C3M	MMP-9 mediated degradation of collagen type III	[47]
C4M	MMP-2, -9, -12 mediated degradation of collagen type IV	[48]
Bone turnover		
OC	Osteocalcin	[41]
CTX-I	C-terminal telopeptide of collagen type I	[42]

*MMP* Matrix-metalloproteinase

### Statistical analysis

Sample size calculation has been described previously [29]. Analyses were performed in MedCalc (version 14.8.1) and GraphPad Prism (version 8). A *p* value below 0.05 was considered statistically significant. Baseline characteristics of healthy individuals, placebo and n-3 PUFA-treated patients were presented as mean ± standard deviation (SD) for continuous variables and number (frequency) for categorical variables. Differences between the groups were calculated using Pearson’s Chi-square for categorical variables and ANOVA (parametric) or Kruskal–Wallis test (non-parametric) for continuous variables. An unpaired *t* test was used to calculate statistical differences between PsA patients and healthy individuals, when data passed the Bartlett’s variation test. If data did not pass, a non-parametric Wilcoxon–Mann–Whitney *t* test was used. A paired *t* test was used for evaluation of treatment at baseline and after 24 weeks. Spearman’s correlations were used to analyse the associations between the biomarker and clinical feature at baseline and after 24 weeks.

## Results

### Demographics

**Fig. 1:**
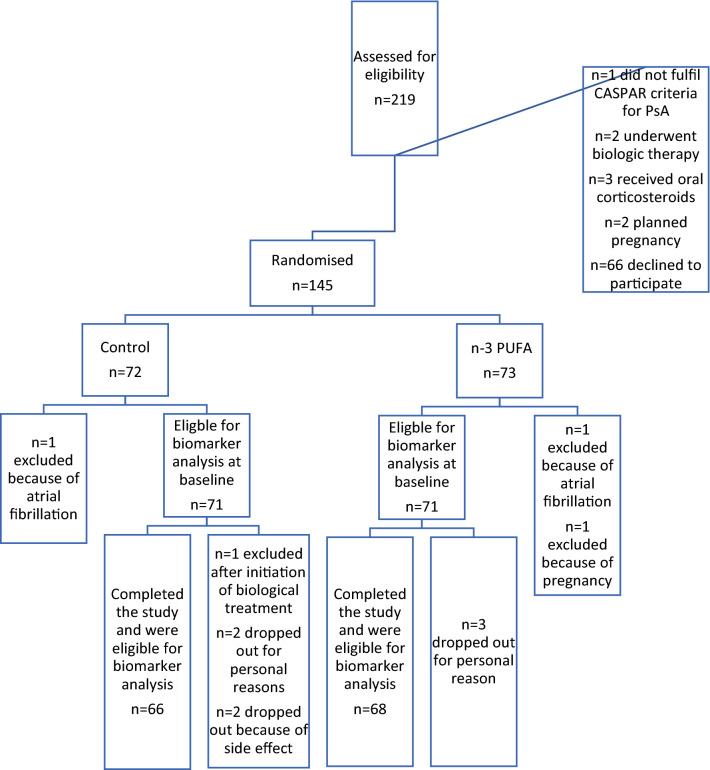
Flowchart of the trial

A total of 142 patients with PsA, 71 patients in the n-3 PUFA group and 71 patients in the placebo group (Fig. 1) and 57 healthy individuals were included in this study. Patient demographics and clinical characterization for the intervention group were compared to baseline characteristics, disease activity scores, and biomarker levels (Table 2) without significant difference and the excluded patients did not differ from those who completed the trial.

**Table 2:** Demographics

Variable	Controls *n* = 57	Placebo *n* = 71	PUFA *n* = 71	*p* value
Mean age (SD)	39.6 (11.8)	50.7 (11.5)	53.0 (11.4)	< 0.001
Gender, female (%)	22 (37%)	43 (60.6)	40 (55.6)	0.038
PsA duration	N/A	13.4 (8.9)	13.4 (10.0)	0.983
BMI (kg/m^2^)	N/A	28.0 (5.0)	28.6 (5.8)	0.529
Smoking, *n* (%)				
Non-smoker	N/A	41 (57.7)	36 (50.0)	0.164
Former smoker		14 (19.7)	27 (37.5)	
Current smoker		16 (22.5)	9 (12.5)	
SJC	N/A	0.8 (2.0)	0.6 (1.9)	0.420
TJC	N/A	4.0 (5.9)	5.1 (9.6)	0.424
ASDAS	N/A	2.3 (1.1)	2.0 (0.9)	0.038
BASDAI	N/A	38.1 (26.3)	32.6 (24.1)	0.199
BASMI	N/A	1.1 (4.0)	1.5 (7.3)	0.669
DAS-28	N/A	2.7 (0.9)	2.5 (0.9)	0.188
LEI	N/A	1.3 (1.6)	1.3 (1.7)	0.802
PASI	N/A	2.3 (4.0)	2.2 (3.0)	0.897
SPARCC	N/A	2.7 (3.3)	2.5 (3.3)	0.703
CRP (mg/L)	N/A	6.1 (7.7)	4.6 (4.2)	0.149
VAS pain	N/A	37.0 (24.3)	29.80 (24.0)	0.085
NSAID use *n* (%)	N/A	32 (45.1)	40 (55.6)	0.746
DMARD use *n* (%)	N/A	50 (70.4)	57 (79.2)	0.136
Documented coronary heart disease, *n* (%)	N/A	4 (5.6)	4 (5.6)	1.000
Hypertension, *n* (%)	N/A	20 (28.2)	21 (29.2)	0.813
Hypercholesterolemia, *n* (%)	N/A	11 (15.5)	18 (25.0)	0.147
Total cholesterol, (mmol/l)	N/A	4.8 (0.8)	5.0 (1.0)	0.196
Systolic BP, (mmHg)	N/A	134.3 (19.2)	137.8 (18.0)	0.275
Diastolic BP, (mmHg)	N/A	82.0 (12.3)	82.7 (10.9)	0.699

*SD* standard deviation, *BMI* body mass index, *SJC* swollen joint count, *TJC* tender joint count, *ASDAS* the ankylosing spondylitis disease activity score, *BASDAI* bath ankylosing spondylitis disease activity index, B*A*SMI bath ankylosing spondylitis meterology index, *DAS-28* disease activity score-28 joints, *LEI* Leeds enthesitis index, *PASI* Psoriasis area and severity index, *SPARCC* spondyloarthritis research consortium of canada, *CRP* C-reactive protein, *VAS* visual analounge scale, *NSAID* non steroidal anti-inflammatory drug, *DMARD* disease modifying anti-rheumatic drugs,* N/A* not available

### Associations between the biomarkers and clinical parameters

Associations between clinical parameters and biomarkers are found in Table 3. A negative correlation was found between CTX-I and DAS-28-CRP (*r* = − 0.199, *p* = 0.018), PRO-C3 and TJC (*r* = − 0.267, *p* = 0.01) and OC; CTX-I; PRO-C1 and SJC (*r* = − 0.234, *p* = 0.005; *r* = − 247, *p* = 0.003; *r *= − 0.236,* p* = 0.005, respectively). A positive correlation was found for C3M and SPARCC (*r* = 0.171, *p* = 0.042). No correlation was found for the other clinical measures and ASDAS-CRP, PASI, LEI, HAQ, BASDAI and VAS pain.

**Table 3:** Correlations between biomarkers and clinical scores

Biomarker	ASDAS-CRP	DAS-28-CRP	TJC	PASI	SJC	LEI	SPARCC	HAQ	BASDAI	VAS pain
OC	Ns	Ns	Ns	Ns	*r* = − 0.234 *p* = 0.005	Ns	Ns	Ns	Ns	Ns
CTX-I	Ns		Ns	Ns	*r* = − 0.247 *p* = 0.003	Ns	Ns	Ns	Ns	Ns
PRO-C1	Ns	Ns	Ns	Ns	*r* = − 0.236 *p* = 0.005	Ns	Ns	Ns	Ns	Ns
PRO-C3	Ns	Ns	*r* = − 0.267 *p* = 0.01	Ns	Ns	Ns	Ns	Ns	Ns	Ns
PRO-C4	Ns	Ns	Ns	Ns	Ns	Ns	Ns	Ns	Ns	Ns
C1M	Ns	Ns	Ns	Ns	Ns	Ns	Ns	Ns	Ns	Ns
C3M	Ns	Ns	Ns	Ns	Ns	Ns	*r* = 0.171 *p* = 0.042	Ns	Ns	Ns
C4M	Ns	Ns	Ns	Ns	Ns	Ns	Ns	Ns	Ns	Ns

*Ns* = non significant

### Extracellular matrix turnover

**Fig. 2:**
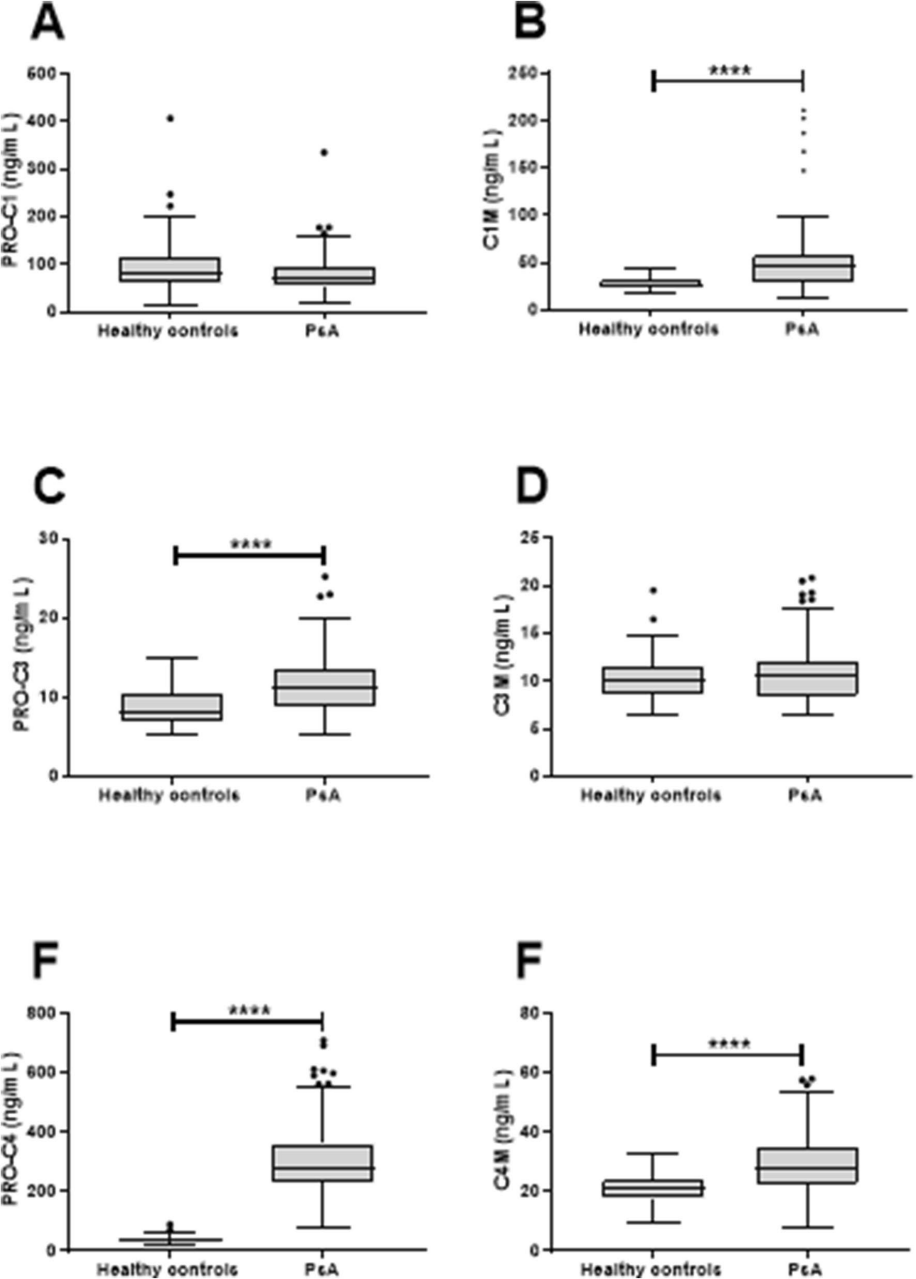
Levels of collagen formation and degradation biomarkers in serum from healthy controls (*n* = 57) and patients with PsA (*n* = 142). **a**. Serum levels of formation of collagen type I (PRO-C1), **b**. Serum levels of degradation of collagen type I (C1M), **c**. Serum levels of formation of collagen type III (PRO-C3), **d**. Serum levels of degradation of collagen type III, **e**. Serum levels of formation of collagen type VI (PRO-C4), **b**. Serum levels of degradation of collagen type IV (C4M). Statistical differences between the healthy controls and PsA patients were calculated using an unpaired *t* test for PRO-C3, and LOG10 transformed PRO-C1, C1M, C3M, PRO-C4 and C4M. Significance threshold was set at *p* < 0.05 and data is presented as Tukey boxplots. Significance levels: ****p < 0.0001

The biomarkers measuring ECM turnover in soft tissue, PRO-C1, measuring collagen type I formation, did not show a difference between the healthy controls and PsA patients (*p* = 0.056, Fig. 2a), while C1M, measuring collagen type I degradation was 56% elevated in patients with PsA (*p* < 0.0001, Fig. 2b). A 76% increase was found for PRO-C3, collagen type III formation in patients with PsA compared to healthy individuals (*p* < 0.0001, Fig. 2c), while no difference was found for the collagen type III degradation marker C3M (*p* = 0.462, Fig. 2d). An increase in both formation and degradation of collagen type IV, reflected by PRO-C4 and C4M, was found (both *p* < 0.0001 and an increase of 792% and 70%, respectively, Fig. 2e and Fig. 2f).

**Fig. 3:**
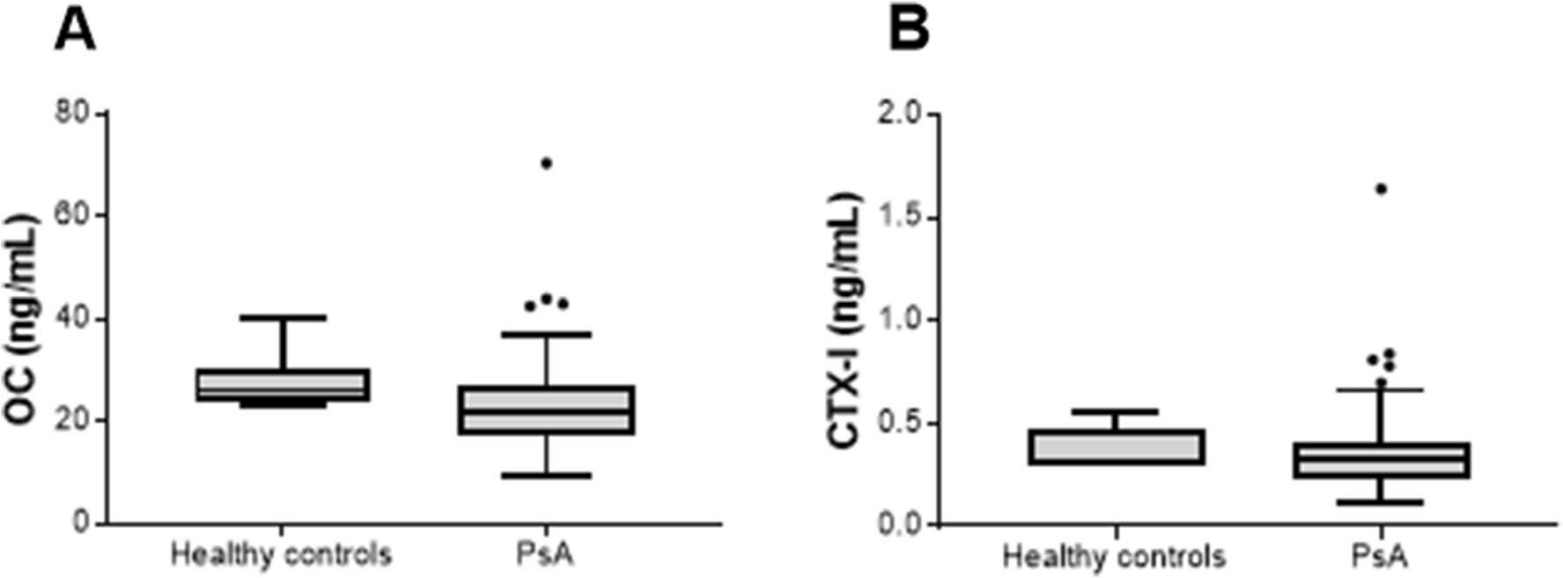
Levels of bone formation and degradation biomarkers in serum from healthy controls (*n* = 6) and patients with PsA (*n* = 142). **a**. Serum levels of bone formation measured by osteocalcin (OC) and **b**. Serum levels of bone degradation measured by CTX-I. Statistical differences between the healthy controls and PsA patients were calculated using an unpaired *t* test for OC, and LOG10 transformed CTX-I. Data is presented as Tukey boxplots

The ECM biomarkers measuring bone formation, OC, and bone degradation, CTX-I, were not statistically different in PsA patients compared to healthy individuals (Fig. 3). A trend towards a decreased level of OC in PsA patients was observed (*p* = 0.06, Fig. 2a

).

### Effect of n-3 PUFA supplementation on bone and tissue turnover biomarkers

**Fig. 4:**
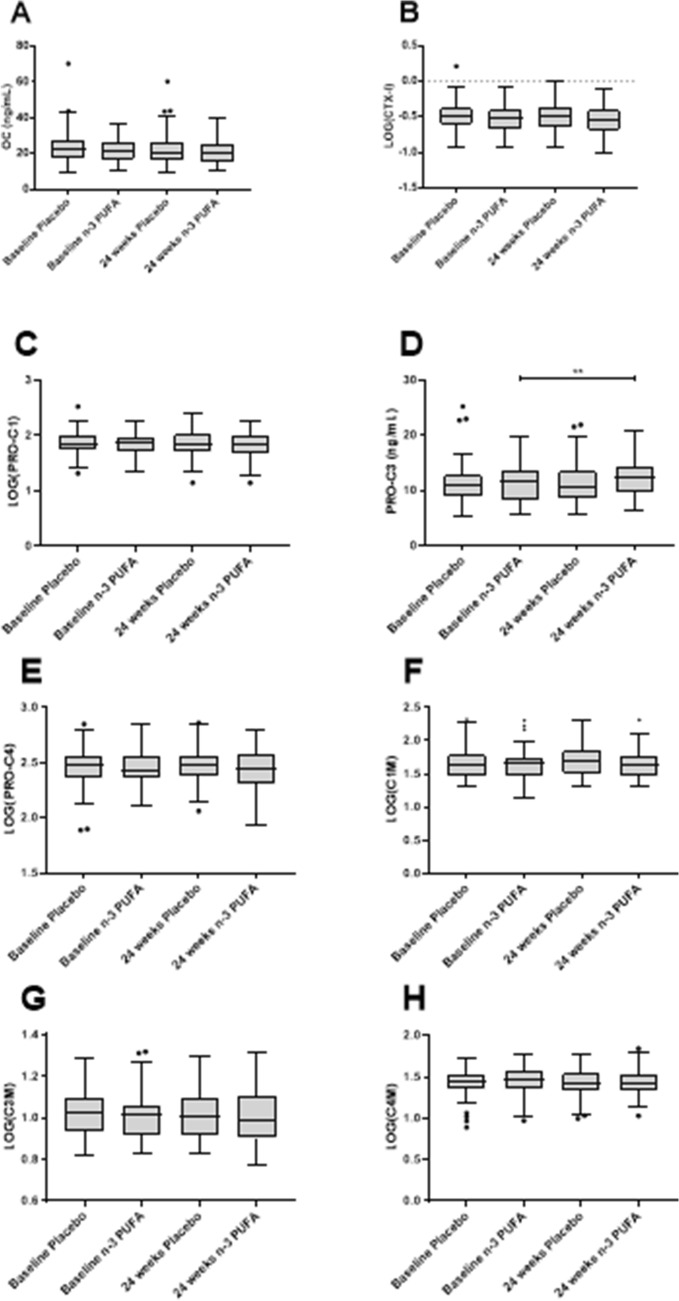
Biomarker levels in placebo (*n* = 57) and n-3 PUFA (*n* = 142) treated patients at baseline and 24 weeks. **a** Serum levels of the bone formation biomarker osteocalcin (OC), **b** Serum levels of the bone degradation biomarker CTX-I, **c** Serum levels of the collagen type I biomarker, PRO-C1, **d** Serum levels of the collagen type III formation biomarker, PRO-C3, **e** Serum levels of the collagen type IV formation biomarker, PRO-C4, **f** Serum levels of the collagen type I degradation biomarker, C1M, **g** Serum levels of the collagen type III degradation biomarker C3M, and h Serum levels of the collagen type IV degradation biomarker, C4M. Statistical differences between the baseline and 24 weeks of follow-up were calculated using a paired *t* test for OC and PRO-C3, and LOG10 transformed CTX-I PRO-C1, C1M, C3M, PRO-C4 and C4M. Significance threshold was set at *p* < 0.05 and data is presented as Tukey boxplots. Significance levels: **p < 0.01

Neither of the bone turnover biomarkers, OC or CTX-I, showed changes in either patients treated with placebo or patients treated with n-3 PUFA for 24 weeks (Fig. 4a and Fig. 4b). PRO-C3 was the only tissue biomarker which was significantly elevated by 7% in patients receiving PUFA-3 (*p* = 0.002, Fig. 4d), none of the other tissue biomarkers showed a significant difference with n-3 PUFA treatment from placebo.

## Discussion

Tissue remodelling is a feature of PsA pathology. Treatment of PsA therefore aims not only to resolve inflammation but also to halt or slow down the ongoing tissue damage. In the present study, we measured ECM turnover biomarkers in healthy individuals, biologic naïve patients with PsA and biologic naïve patients with PsA treated with n-3 PUFA for 24 weeks. To our knowledge, this is the first study assessing tissue neo-epitope biomarkers in a randomised clinical study of patients with PsA. The evaluated tissue biomarkers are anchored in both the interstitial matrix and basement membrane and could therefore potentially be used to describe disease activity in PsA.

N-3 PUFA, especially long-chain n-3 PUFA like eicosapentaenoic acid (EPA) and docosahexaenoic acid (DHA), has shown bone-protective effects in animal models by inhibiting bone resorption and promoting bone formation [35]. Several studies in humans have also been done to assess the effect of n-3 PUFA supplementation on bone health, most of which are done in healthy individuals, some in patients with RA and very few in patients with PsA. Some studies have reported modulation of bone biomarkers after n-3 PUFA intervention [34, 36, 37] while others have reported no effect [38, 39].

Anti-inflammatory and pro-resolving effects of n-3 PUFA have been well-established during the past decades. The underlying mechanisms responsible for n-3 PUFA’s biological effects are mediated by the production of pro-resolving mediators, which have been proposed to modulate and likely resolve inflammatory responses [40]. Therefore, various studies have been carried out to explore therapeutic potential of n-3 PUFA supplementation in inflammatory diseases including inflammatory arthritides [41]. A number of studies have been carried out in patients with RA and other inflammatory diseases that have reported an inverse correlation between n-3 PUFA use and CRP and erythrocyte sedimentation rate [42–44]. In addition to reduced inflammation, n-3 PUFA treatment has also showed a reduced joint pain and NSAID use in patients with RA, and a reduction in use of NSAIDs and paracetamol in patients with PsA [29]. Nevertheless, only a few small studies have examined the effect of n-3 PUFA on clinical outcomes and inflammation in patients with PsA with diverging results [45–47].

In general, several studies have indicated a disturbance in lipids in PsA patients, by an increased serum concentration of triglycerides, cholesterol, LDH and HDL [48, 49]. Saturated fatty acids (myristic acid, palmitic acid, stearic acid, arachidic acid, behenic acid, lognoceric acid) are believed to have pro-inflammatory effects, while MUFA (palmutoleic acid, oleic acid, nervonic acid) and PUFA have shown anti-inflammatory effects [50]. In addition, the compositions of fatty acids, are less important that the overall fatty acid composition [51]. Due to lack of research within the composition of fatty acids in PsA, only speculations on how tissue biomarkers will be modulated dependent on the fatty acid supplement.

In this study, we were unable to demonstrate any changes in biomarkers of bone and tissue turnover after intervention. Only a significant change was found with an increase in PRO-C3 from baseline to 24 weeks, however no relevant clinical finding. The primary outcomes of the clinical trial were not reached and indicated no difference in placebo vs. n-3 PUFA supplement which may describe the lack of changes in the biomarkers [29]. Supplementation with n-3 PUFA for 24 weeks might be too short a period to observe changes and modulation in these biomarkers. Furthermore, patients in this study had a low mean disease activity score of 2.6 (SD = 0.9) at baseline and 75% of the patients received disease-modifying antirheumatic drugs. Thus, we investigated a patient group in remission and results may not apply to patients with a more severely disease activity or patients not receiving DMARDs.

### Limitations and strengths

The present study had some limitations. First, olive oil was used as placebo and while there are reports on the use of olive oil for this purpose in studies investigating the effects of n-3 PUFA [23, 52], it may itself have anti-inflammatory effects owing to its numerous phenol constituents [53]. These effects of olive oil might explain the lack of a significant difference in inflammatory biomarkers between the groups. Second, patients with all disease activity scores were included in the study and they had a low disease activity at baseline (low mean swollen and tender joint count, enthesitis score and PASI) owing to DMARD use in about 75% and NSAID use in 50% of the patients. Third, the effect of n-3 PUFA on clinical outcomes and thereby biomarkers is likely to be small in comparison to DMARDs, thus requiring a larger sample size. Lastly, blood samples were not collected in fasting state for this study, which could have influenced the results, especially CTX-I.

The main strength of the study is the double-blind, randomized, and prospective design comparing subjects which are biologic naïve. Furthermore, the compliance rate was good and only few patients were lost to follow-up.

## Conclusion

Patients with PsA showed an imbalanced ECM turnover compared to healthy individuals. No major differences were found for the biomarkers when the patients were treated with n-3 PUFA for 24 weeks. This indicates that tissue remodelling is occurring in the PsA patients compared to healthy individuals, but the n-3 PUFA treatment for 24 weeks does not have an effect on ECM turnover. However, further long-term studies are required to evaluate the potential effect of n-3 PUFA on PsA.

## Data Availability

The datasets used and/or analysed during the current study are available from the corresponding author on reasonable request.

## Abbreviations

PsA: Psoreatic arthritis
ECM: Extracellular matrix
PUFA: Polyunsaturated fatty acids
PRO-C1: Collagen type I formation
PRO-C3: Collagen type III formation
PRO-C4: Collagen type IV formation
C1M: MMP-mediated collagen type I degradation
C3M: MMP-mediated collagen type III degradation
C4M: MMP-mediated collagen type IV degradation
MMP: Matrix metalloproteinase
OC: Osteocalcin
CTX-I: C-terminal telopeptide of collagen type I
RA: Rheumatoid arthritis
CASPAR: Classification criteria for psoriatic arthritis
TJC: Tender joint count
VAS: Visual analogue scale
HAQ: Health assessment questionnaire
ASDAS: Ankylosing spondylitis disease activity score
BASDAI: Bath ankylosing spondylitis disease activity index
BASMI: Bath ankylosing spondylitis metrology index
LEI: Leeds enthesitis index
SPARCC: Spondyloarthritis research consortium of Canada enthesitis index
PASI: Psoriasis area and severity index
SD: Standard deviation
